# A Clinical Fallacy: Myth of Meningitis-Driven Dural Healing in Post-traumatic Cerebrospinal Fluid Leaks

**DOI:** 10.7759/cureus.89179

**Published:** 2025-07-31

**Authors:** Muath Hussein, Ali Msheik, Amr Rida El Mohamad, Jacinthe Khater, Abdullah Illeyyan, Nasser AlSaad, Abdelnaser Thabet

**Affiliations:** 1 Neurosurgery, Hamad General Hospital, Doha, QAT; 2 Neurosurgery, Hamad Medical Corporation, Doha, QAT; 3 Medical Sciences, Lebanese University Faculty of Medicine, Beirut, LBN

**Keywords:** cerebrospinal fluid leak, dural defect healing, meningitis, skull base fracture, traumatic brain injury

## Abstract

This systematic review evaluates the management of cerebrospinal fluid (CSF) leaks following traumatic skull base fractures and examines the associated risk of post-traumatic meningitis (PTM). It also critically investigates the debated hypothesis that meningitis may promote spontaneous closure of defects in the dura mater through inflammation-induced healing. A comprehensive literature search was performed using PubMed, Scopus, and the Cochrane Library according to Preferred Reporting Items for Systematic Reviews and Meta-Analyses (PRISMA) guidelines. Out of 8,441 records initially screened, 57 full-text articles were reviewed, and 11 studies were included in the final qualitative synthesis. Extracted data included patient demographics, characteristics of CSF leakage, treatment strategies, incidence of meningitis, and clinical outcomes. A total of 216 patients were analyzed, most of whom were young adult males with skull base fractures involving the frontal sinus, ethmoid roof, or cribriform plate. Conservative treatment methods, such as bed rest, head elevation, and blood pressure control, showed success rates ranging from 39.5% to 85%, particularly in middle cranial fossa (MCF) injuries. Surgical repair was ultimately required in 46% of cases, with success rates ranging from 59.3% to 77%. Meningitis developed in 27% of patients, with the highest risk observed in those with delayed or recurrent CSF leakage. The use of prophylactic antibiotics produced inconsistent outcomes, and the recurrence rate was notably higher among those treated conservatively. Traumatic CSF leaks carry a significant risk of infection, especially when diagnosis or treatment is delayed. While isolated reports have suggested that meningitis-induced inflammation might aid in healing, the evidence from this review does not support that claim. Instead, meningitis more frequently impairs healing and increases the likelihood of complications. Prompt identification, individualized treatment planning, and infection prevention are essential to optimize outcomes.

## Introduction and background

Cranial base fractures represent a significant subset of traumatic brain injuries (TBI), accounting for 3.5% to 24% of head trauma cases. These fractures, especially when involving the anterior cranial fossa (ACF), pose a high risk for complications due to the anatomical vulnerability of the skull base. In road traffic accidents, the leading cause of cranial base fractures, the ACF is most frequently affected (70%), followed by the middle (20%) and posterior (5%) fossae. The dura mater in these regions is relatively thin and more prone to tearing, particularly in high-impact injuries. When a dural tear occurs, cerebrospinal fluid (CSF) may leak into adjacent cavities, manifesting as rhinorrhea or otorrhea. These leaks compromise the protective barrier of the central nervous system and expose patients to severe infections such as bacterial meningitis [1,2].

Post-traumatic meningitis (PTM) is a devastating complication of skull base fractures, contributing significantly to morbidity and mortality [2-6]. Mortality associated with PTM has been reported to range from 29% to 57.9%, with risk factors including prolonged external ventricular drainage (EVD), delayed surgical intervention, and extended operative time. In resource-limited settings, delayed access to neurosurgical care further exacerbates this risk.

Pediatric populations, while less frequently affected by skull base fractures, present unique diagnostic and therapeutic challenges. Subtle radiographic findings and nonspecific symptoms may mask CSF leaks, increasing the likelihood of unrecognized fistulas and subsequent meningitis [4,5]. Although many pediatric CSF leaks resolve spontaneously, persistent leaks necessitate timely surgical intervention to prevent infectious sequelae.

A long-standing theory in clinical practice suggests that meningitis may paradoxically aid in the closure of traumatic CSF leaks by stimulating fibroblastic activity and inflammatory adhesion, thereby promoting dural healing. This belief, although not supported by robust clinical evidence, has persisted in part due to anecdotal observations and isolated case reports where CSF leaks appeared to seal following an infectious episode.

However, the spontaneous resolution of traumatic CSF leaks is well-documented, and up to 20% of patients still develop bacterial meningitis, particularly when fractures involve delicate anatomical structures like the cribriform plate [4-7]. Occult vascular injuries and unrecognized dural tears may predispose patients to long-term neurological sequelae. Advanced imaging techniques such as high-resolution CT and MRI with constructive interference in steady state (CISS) 3D sequences are essential for detecting subtle defects not apparent in conventional imaging.

Surgical repair is often indicated when leaks persist beyond the early post-injury phase or are associated with large dural defects. Endonasal and transcranial approaches are employed based on defect location and complexity. However, the timing of surgical repair remains debated. Some advocate for early closure to minimize infection risk, while others suggest a delayed approach, allowing for spontaneous closure within the first 72 hours [4,5,8]. The use of prophylactic antibiotics also remains controversial; while intended to prevent meningitis, their overuse may contribute to antibiotic resistance without clear evidence of benefit [3,9].

Given the variability in patient presentation, fracture pattern, and available resources, the management of traumatic CSF leaks and associated meningitis remains complex. This systematic review aims to critically examine diagnostic strategies, treatment modalities, and outcomes in patients with post-traumatic CSF leaks, while specifically assessing the validity of the theory that meningitis facilitates healing and exploring its actual impact on clinical prognosis.

## Review

Methods

This review was conducted by the Preferred Reporting Items for Systematic Reviews and Meta-Analyses (PRISMA) guidelines. A comprehensive literature search was performed across three electronic databases: PubMed, Scopus, and the Cochrane Library from 2000 to 2025. The search aimed to identify studies addressing post-traumatic CSF leaks, their management strategies, and the associated risk of meningitis. The search terms included combinations of “cerebrospinal fluid leak,” “CSF rhinorrhea,” “skull base fracture,” “post-traumatic meningitis,” “traumatic brain injury,” “conservative treatment,” “surgical repair,” and related MeSH terms. The full search strategy, including databases, Boolean operators, filters, and applied limits, is available in Appendix A.

Eligible studies were those that reported clinical outcomes in patients with traumatic CSF leaks, with or without the development of meningitis. Inclusion criteria encompassed observational studies, systematic reviews, case series, and case reports involving adult or pediatric populations. Studies focusing on non-traumatic or spontaneous CSF leaks, animal models, or articles not available in English were excluded. Studies were also excluded if outcome data were missing or unclear.

Data were extracted independently using a standardized form, capturing key information such as study design, population demographics, etiology and anatomical location of CSF leaks, diagnostic techniques (β₂-transferrin testing, CT cisternography, or MRI), treatment modalities (conservative versus surgical), antibiotic usage, timing and incidence of meningitis, and clinical outcomes including recurrence rates and duration of follow-up. Due to heterogeneity in study designs, methodologies, and outcome definitions, a meta-analysis was not performed. Instead, a narrative synthesis was undertaken to summarize the key findings, identify trends, and explore areas of agreement and discrepancy across the selected studies.

No formal risk of bias assessment was performed due to the narrative nature of this review.

Results

**Figure 1:**
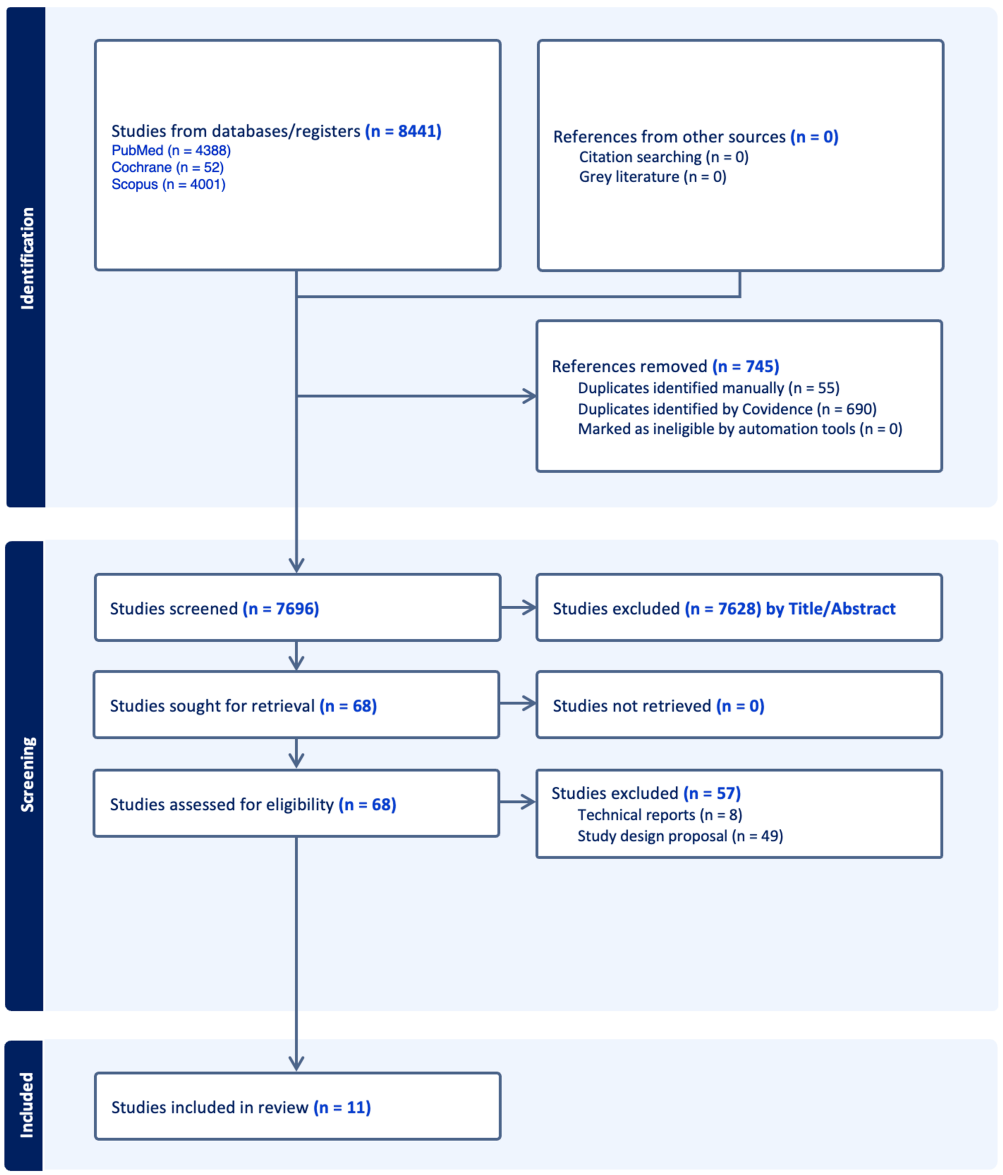
A PRISMA flowchart outlining the study selection process PRISMA: Preferred Reporting Items for Systematic Reviews and Meta-Analyses

The initial search yielded a total of 8,441 records. After removing 745 duplicates, 7,696 titles and abstracts were screened (Figure 1). Following this screening process, 7,628 records were excluded based on irrelevance to the study objectives or failure to meet the inclusion criteria. Full-text retrieval was attempted for 68 articles that were assessed for eligibility, and 11 studies met all criteria and were included in the qualitative synthesis (Table 1).

**Table 1:** Summary of included studies on post-traumatic cerebrospinal fluid leaks and associated risk of meningitis RR: relative risk; CSF: cerebrospinal fluid; aOR: adjusted odds ratio; ACF: anterior cranial fossa; ED: emergency department; NIS: Nationwide Inpatient Sample; TBI: traumatic brain injury

Author (Year)	Study Type	Sample Size	CSF Leak Present	Surgical Factors Evaluated	Meningitis Incidence	Independent Predictors of Meningitis	Notes
Artha et al. (2022) [10]	Systematic review and meta-analysis	15 studies	Yes (analyzed as a subgroup)	Not applicable	RR 16.18 (CSF leak); RR 2.59 (anterior fracture)	CSF leak, anterior cranial base fracture	RR for antibiotics: 0.67 (not statistically significant)
Katayama et al. (2021) [11]	Retrospective cohort (12 years)	60,390	Yes (CSF fistula analyzed)	Burr hole surgery, craniectomy, ED surgery	0.5% (284 cases)	CSF fistula (aOR 8.34), ED burr hole (aOR 1.67), craniectomy (aOR 1.56)	Nationwide trauma database
Leibu et al. (2017) [12]	Retrospective study	47 children	Yes (rhinorrhea/otorrhea in 17%)	Not specified	One case (2.1%)	Not reported	CSF leaks resolved with follow-up; no recurrence
Phang et al. (2016) [13]	Retrospective cohort	50 patients	Yes (post-traumatic skull base fracture)	Endoscopic vs. open surgical repair	Six patients (12%)	Persistent CSF leak	Delayed surgery was associated with recurrent leaks and meningitis
Yellinek et al. (2015) [14]	Retrospective chart review	107 patients	Yes (16/107)	Observation, lumbar drain, acetazolamide, surgery	4/16 (25%)	Not specified	Spontaneous resolution occurred in 75%; close monitoring advised
Yaldiz et al. (2015) [15]	Retrospective case series	13 patients	Yes (all with recurrent meningitis)	Intracranial repair with/without fibrin glue	100% (recurrent)	Large dural/bone defect, brain abscess, craniectomy defect	Success after first surgery: 77%; fibrin glue use was not associated with better outcome
Schoentgen et al. (2013) [16]	Retrospective cohort (10-year review)	40 patients	Yes (100%)	Surgical repair vs. observation; endonasal vs. neurosurgical approach	10% overall; significantly higher with observation (p = 0.0003)	Non-surgical management (wait and see)	Surgery reduced recurrence; anosmia was more frequent with the neurosurgical approach
Sherif et al. (2011) [17]	Prospective observational with algorithmic management	138 patients	Yes (post-traumatic ACF fractures)	Initial conservative management; surgery after 5 days if persistent	1.9% postoperative; 4.3% preoperative	Persistent leak beyond five days	Intradural bicoronal approach preferred; conservative first, then surgery
Sonig et al. (2012) [18]	Nationwide database (NIS) analysis	60,039 TBI patients	0.55% had posttraumatic CSF fistula	Not reported in detail	13.5% in patients with CSF fistula vs. 0.5% without	Presence of CSF fistula (significant predictor)	CSF leak was strongly correlated with increased risk of meningitis
Friedman et al. (2001) [19]	Retrospective case series (15-year single-center review)	51 patients	Yes (all had leaks >24 hours post trauma)	Timing of surgery, antibiotic prophylaxis, and surgical technique	27.5% overall; 14% in clinically evident leaks	Persistent CSF leak, occult presentation	53% spontaneous resolution; 47% required surgery; antibiotic prophylaxis halved the meningitis rate
Bernal-Sprekelsen et al. (2000) [20]	Retrospective cohort (tertiary center)	27 patients with CSF fistula from 1036 TBI cases	Yes (2.6% of TBI cases)	Conservative vs. transcranial repair	33.3% (nine patients); occurred up to nine years post trauma	Persistent or missed CSF fistula, inadequate separation between cranial cavity and sinonasal tract	No statistical difference in meningitis rates between conservative (29%) and surgical groups (40%)

A total of three observational studies were included in this review, comprising two retrospective cohort studies and one prospective observational study (Table 2). No randomized controlled trials were identified, underscoring the limited availability of high-level evidence in the management of post-traumatic CSF leaks.

**Table 2:** Detailed characteristics of observational studies on post-traumatic CSF leaks and meningitis CSF: cerebrospinal fluid; avg: average; RTA: road travel accident; CT: computed tomography; MRI: magnetic resonance imaging; ACF: anterior cranial fossa; MCF: middle cranial fossa

Study	Sample Size	Demographics	Etiology and Fracture Site	Diagnostic Tools	Conservative Management	Surgical Management	Meningitis Incidence	Complications and Outcomes
Sherif et al. (2011) [17]	138	88.9% male; average age ~34 years old	Anterior skull base fractures (cribriform plate, ethmoid roof); mostly RTAs and falls	Clinical signs, Beta-2-transferrin, CT/MRI	39.5% attempted; successful in MCF leaks	For persistent/large leaks, endoscopic and transcranial repairs	27%, higher in late or recurrent leaks	41% recurrence (conservative), 18% (surgical); follow-up up to 6 years
Friedman et al. (2001) [19]	51	77% male; avg age ~35 years old	ACF and MCF fractures; rhinorrhea and otorrhea	Clinical, imaging, Beta-2-transferrin	Initial strategy: 54% success	Duraplasty with fascia lata and fibrin glue	33%; occurred 1-3 years post-trauma	Brain abscess, pneumocephalus; some healing linked to inflammation
Bernal-Sprekelsen et al. (2000) [20]	27	Majority male; avg age ~30 years old	ACF fractures; mostly traumatic origin	CT cisternography, Beta-2-transferrin	Limited use; low success	Endoscopic with fascia lata; open surgery for large lesions	44%; frequent in conservatively treated cases	Multiple reoperations; early surgery recommended

Collectively, these studies reported 216 patients: 138 from Sherif et al. (2011), 51 from Friedman et al. (2001), and 27 from Bernal-Sprekelsen et al. (2000) [17,19,20]. Patient demographics showed that the majority of individuals were male (77% to 88.9%), with a mean age between 30 and 40 years. CSF leaks were predominantly traumatic in origin, arising from motor vehicle collisions, falls, or penetrating trauma such as gunshot wounds.

Demographics and Etiology

Patient demographics across the studies showed that most individuals were male (ranging from 77% to 88.9%) with a mean age between 30 and 40 years. The etiology of CSF leaks was predominantly traumatic, resulting from motor vehicle collisions, falls, or penetrating injuries such as gunshot wounds [17,19,20]. Fractures of the anterior skull, particularly involving the frontal sinus, ethmoid roof, and cribriform plate, were most commonly associated with CSF fistulas, while a minority involved the middle cranial fossa (MCF) or petrous bone.

Spontaneous vs Surgical Resolution

Approximately 54% of patients experienced spontaneous resolution of their CSF leaks, while 46% required surgical intervention [10-12]. Diagnosis was typically based on clinical presentation, β₂-transferrin testing, and imaging such as CT cisternography or MRI. Surgical procedures included duraplasty with autologous fascia lata grafts, occasionally augmented with fibrin glue [20]. Extensive lesions required bone grafts or vascularized flaps. Endoscopic repair was favored for ACF leaks, while larger or more complex defects were managed via open transcranial approaches.

Conservative Management

Conservative treatment, which included bed rest, head elevation, and blood pressure management, was primarily used in less severe cases. Reported success rates for conservative management ranged from 39.5% to 85%, with MCF fractures responding more favorably (60%) than ACF fractures (26.4%) [20]. In patients with large dural defects, encephaloceles, or persistent leakage beyond seven days, surgery was typically required [20].

Complications

Meningitis developed in approximately 27% of cases, with onset ranging from several days to years post trauma [17,19,20]. Patients with delayed or occult CSF leaks were particularly vulnerable. Recurrent CSF leakage was documented in 18% of surgically treated patients and in 41% of those managed conservatively, although intergroup comparisons did not consistently reach statistical significance. Other complications included pneumocephalus, brain abscesses, and the need for reoperation [20].

Antibiotics and Prophylaxis

The use of prophylactic antibiotics varied, with some studies reporting a potential reduction in infection rates, while others showed no clear benefit. One study noted a lower infection rate among those receiving prophylactic antibiotics, though the effect did not reach statistical significance [19]. Concerns regarding long-term resistance and disruption of normal flora were highlighted in broader systematic reviews [10,11].

Surgical Outcomes and Follow-up

Surgical outcomes were generally favorable, with success rates of 77% to 100% and low postoperative complication rates when interventions were timely [17,19,20]. Follow-up periods ranged from six months to six years, with most patients achieving durable closure. Redo surgeries or cerebrospinal diversion procedures, such as lumboperitoneal shunting, were occasionally required [17,19,20].

Meningitis and Healing

Although not the primary outcome in most studies, the potential role of meningitis in modulating the healing of dural defects was occasionally noted. In rare cases, local inflammatory responses associated with meningitis may have promoted fibroblastic activity and scarring, possibly contributing to spontaneous leak closure [13,14,20]. However, this effect was inconsistently reported and not well-documented. In contrast, most evidence suggests that meningitis adversely affects dural healing by damaging meningeal tissues, disrupting initial repair, and predisposing to recurrent leakage [9-11,20].

Economic and Institutional Considerations

From an economic perspective, the presence of CSF leaks and subsequent meningitis significantly increased hospitalization costs, particularly in patients experiencing delayed diagnosis, recurrent leakage, or prolonged intensive care unit stays [12,19].

Discussion

Demographics and Etiology

The epidemiology of post-traumatic CSF leaks reflects the pattern of cranial base fractures, with ACF fractures being the most common, particularly following road traffic accidents. Pediatric cases were less frequently reported but posed greater diagnostic challenges due to subtle clinical signs and incomplete skull base ossification. Males in their 30s to 50s represented the most commonly affected demographic. The included studies consistently associated high-impact blunt trauma with the development of CSF fistulas, often accompanied by dural and osseous disruption.

Spontaneous vs. Surgical Resolution

Spontaneous resolution of CSF leaks was reported in 50% to 85% of conservatively managed cases, depending on the fracture location and timing of diagnosis. ACF leaks showed the lowest spontaneous closure rates, likely due to the fragility of the cribriform plate and persistent nasal pressure gradients. Table 2 summarizes outcomes by fracture location, showing higher surgical conversion rates in ACF compared to MCF fractures. Conservative management strategies, including bed rest, head elevation, and blood pressure control, were frequently successful in the acute setting but carried an increased risk of delayed complications if leaks persisted beyond five to seven days.

Surgical repair was indicated in cases with persistent leaks, large dural defects, or associated encephaloceles. Techniques varied based on location and complexity, including endoscopic endonasal repair (commonly for ACF defects), transcranial approaches, and use of autologous grafts such as fascia lata. Table 3 provides a summary of repair techniques and success rates across studies.

**Table 3:** Summary of surgical and conservative CSF leak repair strategies used in patients with skull base fractures, highlighting associated meningitis rates and outcomes across multiple studies. RR: relative risk; OR: odds ratio; CSF: cerebrospinal fluid; ABX: antibiotics

Study	Number of patients	Repair technique	Success rate	Meningitis incidence
Artha et al. (2022) [10]	Multiple studies (15 included)	Conservative vs. surgical	Not quantified	RR 1.77 surgical group vs. conservative
Katayama et al. (2021) [11]	60,390 patients	Various (burr hole, decompression, etc.)	Not available	0.5%; OR 3.3 with CSF leak
Leibu et al. (2017) [12]	196 children	Surgery, spinal drainage, conservative	Spontaneous resolution in 63% of CSF leaks	2/196 (1%)
Phang et al. (2016) [13]	Not stated	Conservative, then surgery if needed	Most resolved without surgery	Not stated
Yellinek et al. (2014) [14]	107 patients	Three underwent surgery	Not quantified	4/107 (3.7%)
Yaldiz et al. (2015) [15]	13 patients	Intradural, fascia lata graft ¬± fibrin glue	Not quantified; 3 had large dural defects	All had a recurrent meningitis history
Schoentgen et al. (2013) [16]	40 patients	Endonasal (14), neurosurgical (15), conservative (11)	Recurrence 22.5%	10%
Sherif et al. (2012) [17]	138 patients	Lumbodorsal, intradural repair, delayed surgery	Leak resolved in all; recurrence 1.9%	1.90%
Sonig et al. (2012) [18]	382,267 patients	Not described (database study)	Not applicable	0.2%; OR 22.8 with CSF rhinorrhea
Friedman et al. (2001) [19]	51 patients	Various; 23 had surgery	Surgery required in 47%, 13% redo	10% with ABX, 21% without
Bernal-Sprekelsen et al. (2000) [20]	27 patients	Transcranial (10), conservative (17)	Not quantified	40% transcranial, 29% conservative

Management Strategies and Outcomes

Initial management typically followed a stepwise approach, beginning with conservative measures in early or low-flow leaks. Success rates approached 85% in MCF fractures, with lower rates in ACF cases [13,17,19]. When leaks persisted beyond one week, particularly in patients with identifiable dural gaps or pneumocephalus, surgical repair was performed using tailored approaches based on anatomical location [14,15,20]. Multimodal strategies, including the use of intrathecal fluorescein and intraoperative endoscopy, enhanced intraoperative localization and closure.

Risk and Timing of Meningitis

PTM remains a serious and prevalent complication, with incidence rates ranging from 0.2% to 88% depending on trauma severity, leak duration, and healthcare setting [17,19,20]. Table 4 outlines the timing of meningitis onset in relation to trauma and leak detection. In many cases, meningitis occurred days to years after the initial insult, with delayed presentations often tied to missed or recurrent leaks. Katayama et al. noted that prolonged external ventricular drainage, delays in neurosurgical intervention, and logistical barriers such as interhospital transfer were key contributors to meningitis risk [11].

**Table 4:** Overview of the temporal relationship between initial head trauma, CSF leak recognition, and onset of meningitis, differentiating between acute, delayed, and recurrent presentations as reported in the literature. CSF: cerebrospinal fluid; OR: odds ratio

Study	Timing of meningitis onset	Relation to leak detection
Artha et al. (2022) [10]	Acute, higher in the surgical than the conservative group	The surgical treatment group had a higher meningitis rate
Katayama et al. (2021) [11]	During hospitalization (acute)	Detected CSF fistula increased risk (OR 3.3)
Leibu et al. (2017) [12]	Two cases during initial hospitalization	CSF leak present in 28%, meningitis in 2%
Phang et al. (2016) [13]	Not specified; mainly early complications	Conservative first-line; leak persistence not fully clarified
Yellinek et al. (2015) [14]	Acute phase (four patients)	CSF leak was clinically evident in all cases
Yaldiz et al. (2015) [15]	Recurrent, timing not precisely stated	Recurrent meningitis was tied to large defects
Schoentgen et al. (2013) [16]	Postoperative (10% of cases)	The leak was detected pre- or postoperatively in all cases
Sherif et al. (2011) [17]	Postoperative (within one year)	Managed with an algorithm post leak detection
Sonig et al. (2012) [18]	Acute (mean LOS: 6.1 days)	CSF rhinorrhea (OR 22.8), CSF otorrhea (OR 9.2)
Friedman et al. (2001) [19]	Average 6.5 years pos trauma (occult leaks)	Most cases had persistent leaks >24 hours
Bernal-Sprekelsen et al. (2000) [20]	Early and delayed, up to decades post trauma	Detected leaks associated with both early and late meningitis

Meningitis and Healing Dynamics

One of the novel aspects explored in this review is the controversial hypothesis that meningitis may facilitate the spontaneous closure of dural defects through inflammation-induced healing. Anecdotal reports and isolated case series suggest that the intense inflammatory response may stimulate fibroblastic proliferation and collagen deposition, mechanisms that could, in theory, promote scar formation and seal CSF leaks [15,19]. These cases often describe spontaneous leak resolution in the aftermath of infection, though they are typically retrospective and lack robust mechanistic data.

Conversely, the majority of studies reported that meningitis worsens clinical outcomes. Infections were frequently associated with necrosis of meningeal tissues, disruption of healing planes, and higher recurrence of CSF leakage [17,20]. Additionally, overlapping symptoms between infection and leak recurrence often led to delayed diagnosis and management. Overall, while the inflammatory hypothesis is biologically intriguing, the current weight of evidence supports a predominantly detrimental role for meningitis in healing dynamics. Further basic science research is needed to delineate which immune pathways may be beneficial versus pathological.

Limitations of the Literature

The majority of the included studies were retrospective and lacked the methodological rigor of randomized controlled trials. Heterogeneity in study design, patient populations, diagnostic tools, and treatment modalities made direct comparisons challenging. Additionally, few studies reported long-term neurocognitive outcomes or standardized quality-of-life assessments post-repair, which limits the broader understanding of patient recovery trajectories. Nonetheless, by synthesizing available evidence across diverse clinical contexts, this review helps to clarify prevailing trends, highlight consistent findings, and identify areas where future high-quality research is most urgently needed.

Implications for Practice and Future Research

Given the observed infection risk and potential for delayed presentation, all patients with skull base trauma should undergo thorough evaluation for CSF leakage, using high-resolution imaging and β₂-transferrin analysis when clinical suspicion arises. Where feasible, early surgical repair may reduce the risk of meningitis and improve long-term outcomes. Future prospective studies are needed to better define the optimal timing for intervention, evaluate the utility of prophylactic antibiotics, and explore the biological mechanisms by which infection may modulate tissue repair at the site of dural defects. In particular, multicenter prospective trials are essential to establish standardized management protocols, while basic science investigations should focus on elucidating the inflammatory and fibroblastic pathways involved in infection-induced dural healing, to separate anecdotal belief from biological plausibility and guide evidence-based care.

## Conclusions

This review examined post-traumatic CSF leaks, with a focus on treatment strategies, the timing and occurrence of meningitis, and the controversial hypothesis that meningitis may facilitate the spontaneous closure of dural defects. The majority of leaks were associated with skull base fractures following traumatic injury. While initial conservative management, including bed rest, head elevation, and blood pressure control, was effective in selected cases, many patients eventually required surgical intervention. Surgical approaches, whether transcranial or endoscopic, were most effective when tailored to the anatomical characteristics of the defect.

Meningitis was more frequently observed in cases involving delayed or recurrent CSF leakage. Although some reports suggested that the inflammatory response linked to meningitis might facilitate healing through scar tissue formation, this was not consistently supported. On the contrary, the predominant evidence indicated that meningitis often impeded healing, elevated the risk of complications, and increased the likelihood of requiring further surgical intervention. Therefore, this review does not substantiate the notion that meningitis plays a beneficial role in the spontaneous resolution of dural defects. Timely diagnosis, individualized treatment planning, and diligent long-term monitoring are essential for the effective management of post-traumatic CSF leaks, with infection prevention remaining a cornerstone of improved patient care. By synthesizing current evidence on outcomes and management strategies, this review may also inform future clinical protocols and support more judicious antibiotic stewardship in cases where the role of prophylaxis remains uncertain.

## Appendices

Appendix A

Full Search Strategy and Filters

Databases searched: PubMed (MEDLINE), Scopus, and Cochrane Library (CENTRAL)

Timeframe: January 1, 2000 - April 15, 2025

Language: English only

Search Terms and Strategy

PubMed (MEDLINE)

("Cerebrospinal Fluid Leak"[Mesh] OR "CSF leak"[tiab] OR "rhinorrhea"[tiab] OR "otorrhea"[tiab]) AND ("Cranial Fossa, Anterior"[Mesh] OR "skull base fracture"[tiab] OR "basilar skull fracture"[tiab] OR "cranial base fracture"[tiab]) AND ("Meningitis"[Mesh] OR "bacterial meningitis"[tiab] OR "post-traumatic meningitis"[tiab]) AND ("healing"[tiab] OR "fibrosis"[tiab] OR "spontaneous resolution"[tiab] OR "sealing"[tiab])

Filters applied: • Publication Date: 2000/01/01 to 2025/04/15 • Species: Humans • Language: English

Scopus

('cerebrospinal fluid leakage'/exp OR 'csf leak':ti,ab OR 'rhinorrhea':ti,ab OR 'otorrhea':ti,ab) AND ('skull base fracture'/exp OR 'basilar skull fracture':ti,ab OR 'cranial base fracture':ti,ab) AND ('meningitis'/exp OR 'post-traumatic meningitis':ti,ab) AND ('healing':ti,ab OR 'fibrosis':ti,ab OR 'spontaneous resolution':ti,ab OR 'dural sealing':ti,ab)

Limits applied: • Humans • English language • Publication years: 2000 to 2025

Cochrane Library (CENTRAL)

("cerebrospinal fluid leak" OR "CSF rhinorrhea" OR "CSF otorrhea") AND ("skull base fracture" OR "cranial base fracture") AND ("meningitis" OR "bacterial meningitis") AND ("healing" OR "fibrosis" OR "spontaneous resolution")

Filters applied: • Trials only • Publication date range: 2000 to 2025 • Language: English

Notes

• All database searches were conducted between April 10 and April 15, 2025. • Reference lists of eligible articles and relevant reviews were manually screened for additional studies. • Duplicates were removed using EndNote (Clarivate, London, UK) and manual review.
